# Emergency departments in Denmark with a research responsible consultant expect increased research production

**DOI:** 10.1186/1757-7241-21-S2-A25

**Published:** 2013-09-09

**Authors:** Cecilie Markvard Møller, Julie Mackenhauer, Anders Brøns Møllekær, Mikkel Brabrand, Peter Hallas, Hans Kirkegaard

**Affiliations:** 1 Research Center for Emergency Medicine, Aarhus University, Denmark; 2 Department of Internal Medicine, Regionshospitalet Viborg, Denmark; 3 Department of Medicine, Sydvestjysk Sygehus Esbjerg, Denmark; 4 Department of Anesthesia, JMC, Rigshospitalet, Denmark

## Background

Emergency medicine (EM) is not a recognized specialty in Denmark, but an area of competence. In Denmark this is new, and current research production in the Emergency departments (EDs) is limited. As in other areas of clinical medicine, there is a need for research to ensure high quality evidence-based patient care.

The aim of our study was to examine 1) the current research activity in the Danish EDs, 2) the impact of having a research responsible consultant on scientific production, 3) the relationship between production and opinion of the head of department with regards to EM as an area of competence respectively a specialty.

## Methods

A survey was conducted among the 21 ED department heads. Data was collected during September 2012. The respondents were asked for 1) number of publications in 2011, 2) number of expected publications in 2012, 3) whether the department had a research responsible consultant and 4) whether the respondent thought an EM specialty compared to an area of competence would increase research activity.

## Results

Answers were obtained from all 21 EDs. In 2011, 48% (n=10) did not publish any scientific papers while the rest published 1-10 papers. In 2012, 24% (n=5) of the departments did not expect any publications (none have a research responsible consultant) and 57% (n=12) expect 1-10 publications (83% (n=10) have a research responsible consultant). Only one department anticipated more than 10 publications.

52% (n=11) of the departments have a research responsible consultant and they expect an increased scientific production the impending year, in fact these departments all expected publications, while only 20% of the departments without such consultant expected publications (p<0,01, Fisher’s Exact test).

62% (n=13) of the department heads agreed in varying degree that an EM specialty compared to an area of competence would increase research activity, while 14% (n=3) disagreed.

## Conclusion

Research activity in the Danish EDs is limited but expected to increase - more in departments with a research responsible consultant than in EDs without. There is a general believe that establishment of an EM specialty will increase research activity.

